# Evaluation of rest interval following a series of tuck jumps on anaerobic performance^☆^

**DOI:** 10.1016/j.jesf.2024.11.001

**Published:** 2024-11-22

**Authors:** Christopher J. Cleary, Summer B. Cook, Ashley A. Herda

**Affiliations:** a Department of Health, Sport, and Exercise Sciences, University of Kansas Edwards Campus, Overland Park, KS, USA; b Department of Kinesiology, University of New Hampshire, Durham, NH, USA; c Department of Orthopedic Surgery and Sports Medicine, Kansas City, KS, USA

**Keywords:** Wingate, Postactivation, Plyometrics, Ergogenic

## Abstract

**Background/objective:**

This study assessed the influence of rest interval duration after tuck jumps on 10-s Wingate outcomes and countermovement jump height.

**Methods:**

Eighteen resistance trained individuals (males: n = 10, 21.3 ± 3.6 years; females: n = 8, 22.1 ± 2.2 years) volunteered to participate in four sessions: familiarization, 3-min rest interval with no jumps (CON), and two randomized experimental sessions with a rest interval of either 1-min (ER1) or 5-min (ER5) after a series of tuck jumps. Countermovement jump (CMJ) height was assessed at baseline (PRE) and after (POST) the CON, ER1, and ER5 conditions, and 10-s Wingate cycling testing. Wingate relative peak power (RPP) and mean peak power (RMP) were measured. Separate mixed-factorial repeated measures analyses of variance assessed changes across conditions and sex for the Wingate variables and conditions, sex, and time for CMJ height at an alpha of p ≤ 0.05.

**Results:**

RPP and RMP were significantly greater than CON for ER1 by 0.92 ± 0.23 W kg^−1^ and 0.41 ± 0.14 W kg^−1^, respectively, and ER5 by 0.77 ± 0.23 W kg^−1^ and 0.36 ± 0.10 W kg^−1^, respectively. ER1 and ER5 RPP and RMP were similar (p > 0.05). For CMJ height, there was only a main effect for sex as males jumped higher than females by 31.3 % (p = 0.002).

**Conclusion:**

Performing tuck jumps prior to anaerobic exercise may increase performance for up to 5-min

## 1 Introduction

Beyond warmups, athletes and coaches are interested in pre-exercise movements or activities that may translate into acutely enhanced sport performance. Acute performance enhancement strategies that have gained popularity over recent years fall into either the postactivation potentiation (PAP) or postactivation performance enhancement (PAPE) phenomenon.^8^^,^^17^^,^^33^^,^^40^ Both involve enhanced muscular or performance responses as the result of the completion of a conditioning activity. The differences lie in the time course of the accentuated response and the activity or movement that is enhanced.^8^^,^^25^ To measure performance changes that may be attributed to PAPE, the conditioning activity is performed prior to an athletic task (e.g., sprinting, jumping) and it is hypothesized that the performance of the athletic task will be enhanced due to PAPE in comparison to the task's performance without a conditioning activity.^4^^,^^11^

When designing a PAPE or PAP protocol, factors to consider include the conditioning activity and rest interval duration between the conditioning activity and the athletic task.^30^ High-load strength training exercises are commonly implemented as conditioning activities, such as, back squats, deadlifts, or Olympic lift variations^6^^,^^35^ with mixed results.^23^^,^^39^^,^^40^ Additionally, strength training exercises may elicit neuromuscular excitation, considered PAP, with limited effect on athletic movements.^33^^,^^39^^,^^40^ In contrast to strength training exercises, plyometrics require minimal equipment and can be completed anywhere, lending plyometrics to be an attractive modality for performance enhancement prior to competition.^4^^,^^21^ Specifically, tuck jumps are a short duration, repetitive task that involves high eccentric and concentric loading.^12^^,^^16^ With these factors in mind, tuck jumps can be a viable option for use in PAPE protocols. Sharma et al. reported that a plyometric battery of ankle hops, hurdle jumps, and drop jumps followed by a 10-min rest interval decreased subsequent 20-m sprint time.^32^ Conversely, Tsolakis et al. determined that three sets of five tuck jumps had no effect on vertical jump height performed 4-, 8-, and 12-min after the tuck jumps.^37^ These previous studies^32^^,^^37^ had varying outcome tasks where the performance enhancement effects may be differentiated by the duration of the movement, such as a single jump like Tsolaksis et al.^37^ presents versus 20-m distance used in Sharma et al.^32^ Lastly, rest interval duration is a vital factor to consider due to the time-constrained nature of the PAPE response.^7^^,^^11^^,^^31^ If the rest interval is too short, then it has been hypothesized that PAPE may be predominated by fatigue, resulting in an inability to observe enhanced performance.^26^ On the contrary, if a rest interval is too long, the PAPE response may deteriorate by the time the outcome task is performed, limiting performance enhancements. A variety of rest interval durations have been investigated and it is vital to attempt to elucidate the optimal rest intervals to take advantage of the performance enhancement due to PAPE or PAP.

Under laboratory settings, it is easy to control the conditions of an outcome variable to maintain internal validity, but it is critical to best select appropriate tasks that can translate to the field, especially for athletic tasks. The 30-s Wingate has been used to evaluate anaerobic capacity through metrics such as peak and mean power output and fatigue index.^1^ While these metrics are relevant, the applicability of 30-s all-out sprints plus the buildup to maximal cycling revolution speed is not as common in *most* sports. Shorter duration quick bursts of explosive movements, however, are.^33^^,^^41^ The 10-s Wingate can provide the same outcomes in close correlation to many athletic tasks.^33^^,^^41^ Specifically, defensive volleyball sets, charges down a basketball court, 100-m sprint, an acceleration in hockey, or a play in football. Therefore, the utilization of a 10-s Wingate as an outcome measure to assess the potential PAPE effect of a conditioning activity can directly relate to short athletic tasks.^1^^,^^41^ Further, using a 30-s Wingate, high-load back squats have not elicited performance enhancements,^17^ yet high-load deadlifts have been reported to improve performance.^34^ PAPE protocols such as high-load back squats or deadlifts have enhanced 10-s Wingate mean power.^33^ The shorter duration may have more direct applicability with the targeted metrics being evaluated and is a less intimidating ask of participants (10-s vs. 30-second Wingate), specifically, when the two test durations provide similar outcome variables. Additionally, despite the attractiveness of plyometric conditioning activities, no previous studies have investigated plyometric conditioning activities as a PAPE protocol for Wingate test performance and subsequently have not investigated how alterations of rest interval duration impact Wingate performance, specifically 10-s Wingate outcomes. Therefore, the purpose of this study was to assess the impact of rest interval duration after tuck jumps on countermovement jump height and 10-s Wingate test performance in males and females.

## 2 Methods

### 2.1 Study design

**Fig. 1:**
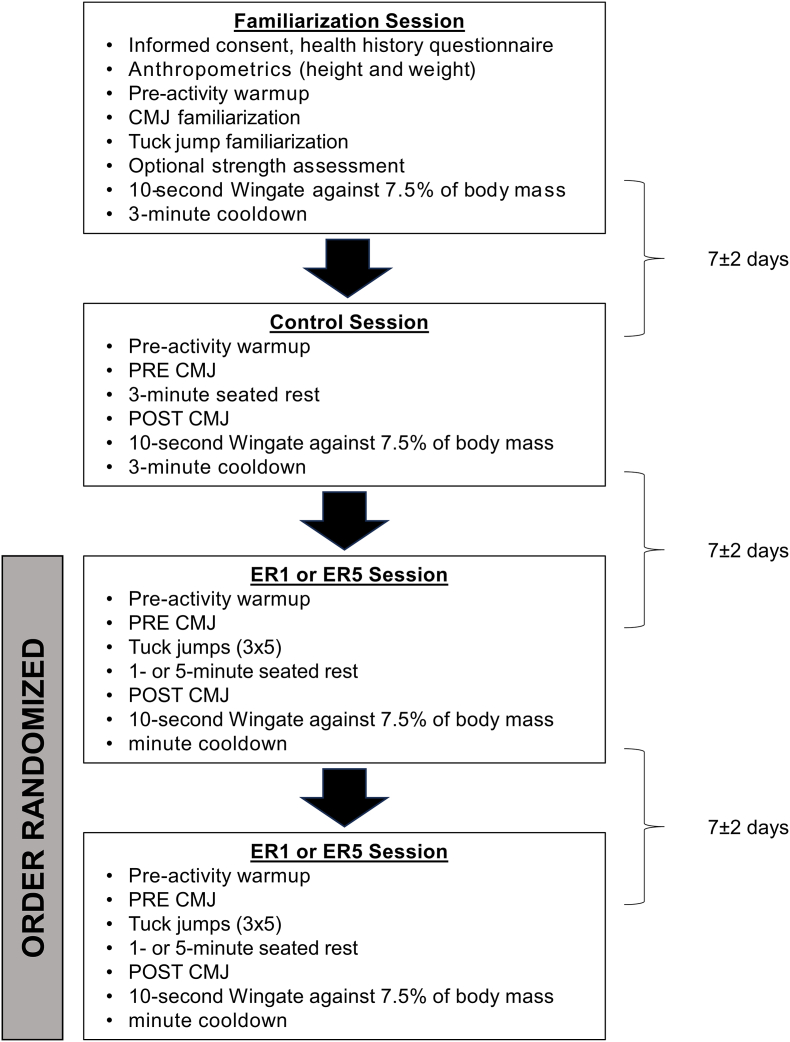
Timeline of study procedures. ER1 = experimental 1-min rest condition, ER5 = experimental 5-min rest condition.

Acute changes in jump height and 10-s Wingate performance were measured using a within-subjects repeated measures design that consisted of four study sessions per participant, each separated by 7 ± 2 days: familiarization, control, and two experimental trials (Fig. 1). Participants were familiarized with all study procedures after completing written forms of informed consent and personal health and exercise history. Body mass was obtained using a digital scale (Model 2084, Toledo scale, Toledo, Ohio, USA) and stature was obtained using a wall-mounted stadiometer (Global Industrial, Inc., DeSoto, TX, USA). Procedures completed during each session included a 5-min warmup, three countermovement vertical jump (CMJ) trials, a series of three sets of five tuck jumps, and a 10-s Wingate test on a cycle ergometer. All participants also had the option to complete a 1-repetition maximum back squat to further determine training status. All procedures were approved in accordance with the Declaration of Helsinki by the (University information blinded for review) Human Research Protection Program (Approval #00146478).

## 3 Participants

An *a priori* power analysis conducted in G∗Power (v 3.1.9.6, G∗Power, Kiel, Germany) determined that a minimum of 16 participants would need to be recruited to achieve 80 % power at an alpha of 0.05 with a moderate to large Cohen's *f* effect size of 0.36 determined from previous research.^10^ A total of 18 participants (10 males, 8 females) volunteered to participate in the present study. Participants were included if they were between the ages of 18–30 years and self-reported being engaged in resistance training 3x/week for the past year. Prospective participants were excluded if they had any history of cardiovascular or nervous system disorders, previous orthopedic or musculoskeletal injuries within the past 6-months, or limitations that they believed would impact their ability to safely complete the study protocols.

## 4 Procedures

### 4.1 Pre-activity warmup and countermovement vertical jump

Each session began with a 5-min warmup on a stationary cycle ergometer (Monark 894E Peak Bike, Monark Exercise, Vansbro, Sweden) at 60–70 rpm against 1.0 kg resistance at the beginning of each session. To assess jump height, standing reach was first measured using a Vertec adjustable vertical jump device (Jump USA, Sunnyvale, CA, USA) following standardized protocols.^5^^,^^19^ The CMJ procedure was explained and demonstrated during the familiarization session. Participants were instructed to squat to a self-selected depth before jumping and reaching overhead to displace the highest vane possible using their dominant arm on the Vertec, following manufacturer guidelines. Participants completed two warmup CMJs followed by a single maximal repetition with 30-s rest between repetitions.

### 4.2 Optional strength assessment

During the familiarization session, to quantify the training status participants could complete an optional 1 repetition maximum (1-RM) back squat assessment. Following guidelines put forth by the National Strength and Conditioning Association, participants performed sets of increasing load and decreasing repetitions with up to five 1-RM trials allowed.^14^ A total of 11 participants performed this optional assessment.

### 4.3 Tuck jump protocol

A tuck jump protocol was utilized as the conditioning activity following similar methods to previous literature^35^^,^^37^ in attempt to elicit PAPE. For all tuck jumps, the participants were provided demonstration and cued to “spend the least amount of time on the ground as possible between repetitions and land in the same spot you took off from”, to limit ground contact time according to the suggestions by Maloney et al.^21^ and jump maximally off both legs while bringing their knees to their chest.^35^^,^^37^ Participants completed three sets of five repetitions with 1-min of rest between each set.

### 4.4 Ten-second wingate

For each 10-s Wingate test, participants cycled maximally against a resistance of 7.5 % of their body mass for 10-s on the Monark ergometer following methods previously validated.^9^^,^^17^^,^^24^ After a 3-s verbal countdown, the participant began cycling maximally and once 100 rpm was reached, integrated software (Monark Anaerobic Test Software, Monark, Sweden) applied the participant's respective resistance to the ergometer flywheel. Throughout the test, the participant was provided strong verbal encouragement while cycling maximally. Following the test, the participant completed a 3-min cool down against light resistance.

Relative peak power (RPP) was determined as the peak power of a 1-s running average during the test in watts per unit of body mass (W∙kg^−1^), relative mean power (RMP) was the mean power in W∙kg^−1^ throughout the entire test, time to peak power (TPP) was defined as the time it took to reach RPP in seconds, and the fatigue index (FI) was determined as the percentage drop in RPP from peak power to the end of the test.

### 4.5 Control session

The control (CON) session was completed approximately 1 week following their familiarization session and prior to the randomized experimental rest (ER) sessions. After the pre-activity warmup, standing reach was again measured followed by two warmup CMJ trials and one maximal CMJ, with the maximal CMJ recorded as the PRE jump height. The participants then rested seated for 3-min, followed by a single maximal CMJ, recorded as the POST jump height immediately prior to the 10-s Wingate test using the previously described procedures. Test-retest reliability measured by the intra-class correlation coefficient (ICC) between the familiarization and CON sessions was high for CMJ (ICC = 0.96) and the 10-s Wingate (ICC = 0.93).

### 4.6 Experimental sessions

The ER sessions followed the same sequence as the CON session (Fig. 1). However, in the randomized ER sessions, the participants performed 3 sets of 5 repetitions of the tuck jumps after the PRE CMJ, rested for either 1-min (ER1) or 5-min (ER5), completed the POST CMJ followed by the 10-s Wingate, and then the cooldown procedures.

### 4.7 Statistical analyses

Independent sample t-tests evaluated sex differences between descriptive statistics (age, stature, body mass, Wingate test load). A three-way 2 × 2 × 3 repeated measures analyses of variance ANOVA [sex (male vs. female) x time (PRE vs. POST) x condition (CON vs. EXP1 vs. EXP5)] was conducted to determine differences in CMJ height. Four separate two-way 2x3 repeated measures ANOVA [sex (male vs. female) x condition (CON vs. EXP1 vs. EXP5)] were conducted to assess differences in RPP, RMP, TPP, and FI for the sprint cycle test data. Significant main effects were followed up with one-way ANOVAs or t-tests collapsed across sex, time, or condition with Bonferroni corrections. Partial eta squared (η_p_^2^) and Cohen's d (*d*) are reported as measures of effect sizes for the ANOVAs and t-tests, respectively. Data were considered significant at p ≤ 0.05 and all analyses were conducted in R Statistical software (v4.2.1, R Core Team, Vienna, Austria) through RStudio (RStudio, Posit Software, PBC, Boston MA, USA).

## 5 Results

Male and female participants were similar in age (Table 1, p = 0.56, *d* = 0.28), yet males were taller (mean difference: 12.2 ± 3.9 cm; p = 0.01, *d* = 1.51) and had greater body mass (19.1 ± 6.4 kg; p = 0.01, *d* = 1.36) than females. The Wingate test load of 7.5 % of body mass was greater for males than females (1.45 ± 0.52 kg, p = 0.01, *d* = 1.39).

**Table 1:** Descriptive characteristics of the participants.

	Males (n = 10)	Females (n = 8)
Age (years)	21.3 ± 3.6	22.1 ± 2.2
Stature (cm)	181.1 ± 5.6	168.9 ± 9.9^a^
Body Mass (kg)	86.6 ± 18.6	67.5 ± 7.1^a^
Wingate Load (kg)	6.5 ± 1.4	5.1 ± 0.5^a^
Relative Back Squat 1-RM (kg∙kg^−1^)	1.54 ± 0.44 kg (n = 6)	1.37 ± 0.16 (n = 5)

Values are presented as mean ± SD.

a difference between sex.

For CMJ height (Table 2), there was no 3-way sex x time × condition interaction (F(2,32) = 0.37, p = 0.69, η_p_^2^ = 0.02), there were no 2-way interactions [sex x time (F(1,16) = 0.39, p = 0.54, η_p_^2^ = 0.02); sex x condition (F(2,32) = 0.76, p = 0.48, η_p_^2^ = 0.05); condition x time (F(2,32) = 0.58, p = 0.56, η_p_^2^ = 0.04)], nor was there a main effect for condition (F(2,32) = 0.44, p = 0.65, η_p_^2^ = 0.03) or time (F(1,16) = 0.26, p = 0.62, η_p_^2^ = 0.02). There was a main effect for sex (F(1,16) = 14.36, p = 0.002, η_p_^2^ = 0.48), where collapsed across time and condition, males had greater CMJ height than females (61.7 ± 9.4 vs. 47.0 ± 6.03 cm, respectively).

**Table 2:** Countermovement jump height in centimeters presented by sex, condition, and time.

	CON		ER1		ER5	
Sex	PRE	POST	PRE	POST	PRE	POST
Males	62.23 ± 8.72^a^ (55.99–68.46)	61.47 ± 8.90^a^ (55.10–67.83)	61.72 ± 10.29^a^ (54.36–69.08)	62.36 ± 9.95^a^ (55.24–69.48)	61.21 ± 10.59^a^ (53.64–68.79)	61.21 ± 10.19^a^ (53.92–68.50)
Females	46.04 ± 6.06 (40.97–51.11)	46.35 ± 5.26 (41.96–50.75)	47.15 ± 6.70 (41.55–52.75)	47.62 ± 7.92 (41.01–54.24)	47.15 ± 5.23 (42.77–51.52)	47.62 ± 6.51 (42.18–53.06)

Values are presented as mean ± SD and (95 % confidence interval).

a difference between sex, collapsed across condition and time; CON: control condition; ER1: experimental 1-min rest condition; ER5: experimental 5-min rest condition; POST: after tuck jumps; PRE: before tuck jumps.

**Fig. 2:**
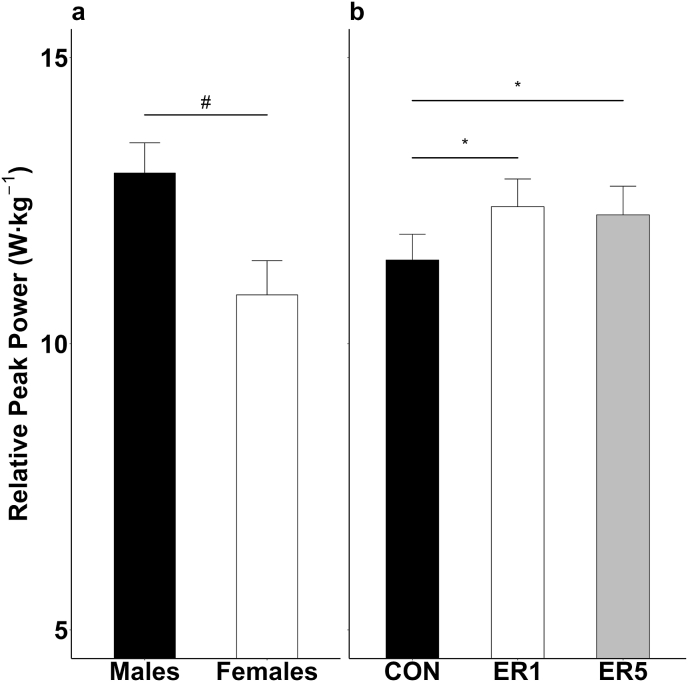
Mean ± SE RPP (a) for males (black) and females (white), collapsed across condition. Mean ± SE RPP (b) for CON (black), ER1 (white), and ER5 (grey), collapsed across sex. Data are presented collapsed across condition (a) and sex (b) as there were no significant 2- or 3-way interactions. ^#^difference between sex, collapsed across conditions; ∗greater than CON, collapsed across sex.

There were no 2-way sex × condition interactions for any of the sprint cycle test variables [RPP (F(2,32) = 0.51, p = 0.61, η_p_^2^ = 0.03), RMP (F(2,32) = 0.05, p = 0.89, η_p_^2^ = 0.01), FI (F(2,32) = 0.18, p = 0.79, η_p_^2^ = 0.02), nor TPP (F(2,32) = 0.49, p = 0.57, η_p_^2^ = 0.04]. For RPP, there was a main effect for sex (Fig. 2A: F(1,16) = 7.12, p = 0.02, η_p_^2^ = 0.31) and condition (Fig. 2B: F(2,32) = 9.59, p < 0.01, η_p_^2^ = 0.38). Collapsed across condition, males (13.0 ± 1.8 W kg^−1^) had greater RPP than females (10.9 ± 1.7 W kg^−1^). Further, in ER1 (12.4 ± 2.1 W kg^−1^; p < 0.01, *d* = 1.40) and ER5 (12.3 ± 2.1 W kg^−1^; p = 0.01, *d* = 1.14), RPP was higher than CON (11.5 ± 1.9 W kg^−1^) with no differences between ER1 and ER5 (p > 0.99, *d* = 0.23).

**Fig. 3:**
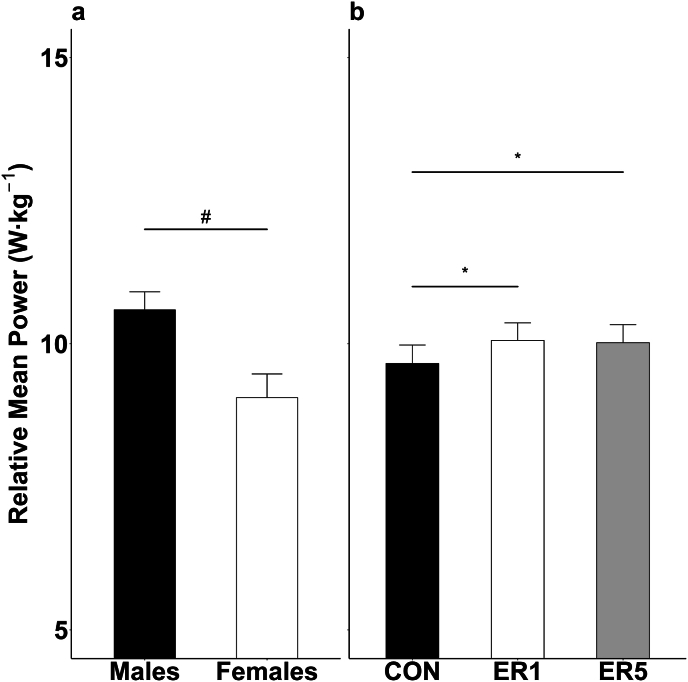
Mean ± SE RMP (a) for males (black) and females (white), collapsed across conditions. Mean ± SE RMP (b) for CON (black), ER1 (white), and ER5 (grey), collapsed across sex. Data presented collapsed across condition (a) and sex (b) as there were no significant 2- or 3-way interactions. ^#^difference between sex, collapsed across conditions; ∗greater than CON, collapsed across sex.

Similarly, for RMP there was a main effect for sex (Fig. 3A: F(1,16) = 9.03, p = 0.008, η_p_^2^ = 0.36) and for condition (Fig. 3B: F(2,32) = 6.62, p = 0.004, η_p_^2^ = 0.29). Males (10.6 ± 1.0 W kg^−1^) had greater RMP than females (9.1 ± 1.2 W kg^−1^) collapsed across condition. Further, RMP in ER1 (10.2 ± 1.2 W kg^−1^; p = 0.028, *d* = 1.1) and ER5 (10.0 ± 1.3 W kg^−1^, p = 0.004, *d* = 0.99) was greater than CON (9.7 ± 1.4 W kg^−1^), while ER1 and ER5 were similar (p > 0.99, *d* = 0.12).

Lastly, there were no main effects for condition (FI: F(2,32) = 1.04, p = 0.37, η_p_^2^ = 0.06 and TPT: F(2,32) = 2.99, p = 0.06, η_p_^2^ = 0.16) or sex (FI: F(1,16) = 0.46, p = 0.51, η_p_^2^ = 0.03 and TPT: F(1,16) = 0.01, p = 0.94, η_p_^2^<0.001) for FI and TPP (Table 3).

**Table 3:** Fatigue index and time to peak power for males and females presented by condition.

	CON		ER1		ER5	
	*Males*	*Females*	*Males*	*Females*	*Males*	*Females*
Fatigue Index (%)	29.32 ± 7.24 (24.13–34.50)	27.67 ± 6.29 (22.41–32.93)	31.48 ± 7.48 (26.13–36.84)	30.48 ± 8.52 (23.36–37.61)	32.31 ± 7.83 (26.71–37.92)	29.08 ± 8.36 (20.09–36.07)
Time to peak power (s)	1.72 ± 0.69 (1.23–2.22)	1.97 ± 1.54 (0.68–3.26)	1.77 ± 1.47 (0.72–2.82)	1.70 ± 0.85 (0.98–2.41)	1.37 ± 0.84 (0.77–1.98)	1.11 ± 0.11 (1.01–1.20)

Values are presented as mean ± SD and (95 % confidence interval). CON: control condition; ER1: experimental 1-min rest condition; ER5: experimental 5-min rest condition.

## 6 Discussion

There has been a recent increase in the use of bodyweight plyometric-type movements as a conditioning activity to elicit PAPE.^3^^,^^4^^,^^35^^,^^37^ The present study evaluated the influence of rest interval duration following a series of tuck jumps on PAPE. Changes in CMJ height and 10-s Wingate test performance with each experimental condition were compared to baseline values without tuck jumps, utilizing the CON condition as baseline for the Wingate and the PRE CMJs as baseline. For the CMJ, there was no PAPE effect as there was no change in CMJ height across time or condition. RPP and RMP were significantly increased in the experimental trials after the tuck jump conditioning activity compared to the CON condition, suggesting a PAPE effect for the Wingate assessment.

Tuck jumps and similar plyometric movements can be performed in any setting, utilize zero equipment, and require only a small space to successfully implement, making them the ideal conditioning activity or pre-exercise movement. These movements as well as other methods described are achievable by high-level and recreational athletes with limited sport-specific experience. A 10-s Wingate test is a continuous, repetitive movement, in a short burst, and can be completed in a safe and controlled manner without lasting effects such as nausea or delayed-onset muscle soreness in comparison to a 30-s Wingate.^41^ Data acquired during this test is applicable to speed and power athletes as it resembles the demands of sport activities to a greater degree than a single repetition of vertical jump task.^24^^,^^33^ In the present study, there were noted enhancements in RPP (ER1: 7.9 % and ER5: 6.1 % greater than CON) and RMP (ER1: 4.3 % and ER5: 3.8 % greater than CON) in response to both rest interval combinations compared to the CON condition. The differences between CON and the experimental trials (ER1 and ER5) may be attributed to various factors that previous investigations have evaluated, including rest or test duration, the conditioning activity utilized, or the relative specificity of the movement and the participant demographics. The enhanced RMP and RRP observed in comparison to the CON trial could be due to physiological mechanisms related to PAPE such as greater muscle temperature or muscle activation^2^ in response to the tuck jump protocol of the present study, Also, the physiological responses may be attributed to the traditional PAP response of enhanced myosin regulatory light chain phosphorylation.^20^^,^^26^ Further, specific to participant demographics, the inclusion of sex-related comparisons in the present study is innovative as PAPE related studies involving sprint cycling primarily have included only males.^17^^,^^33^^,^^34^ The results of the present study indicated no unique responses to the PAPE protocol between males and females despite there being main effects of sex for RMP, RPP, and jump height. These sex-related differences have been extensively reported^22^^,^^27^^,^^38^ and could potentially be due to factors such as muscle mass and hormonal differences.^28^

Despite slight methodological differences in conditioning activities and timing, the present findings are in agreement with other studies.^33^^,^^34^ Smith et al. reported a 6.9 % increase in absolute mean power during a 10-s Wingate 5-min after 10 sets of single 90 % 1-RM back squat movements compared to a control condition.^33^ Additionally, a 10-min rest interval after 5 repetitions at 85 % 1-RM deadlifts enhanced peak power (∼9.0 %) and mean power during the first 5- (∼12.0 %) and 10-s (∼9.0 %) of a 30-s Wingate compared to a control condition.^34^ Further, whole body vibration applied during half squats has elicited performance increases mean and peak power of a 10-s Wingate test after 1-min rest.^10^^,^^29^ Similarly, peak power was also improved for a 15-s Wingate with no changes in mean power compared to a condition without whole body vibration.^29^

Alternatively, 5 back squats at 85 % of 1-RM, and a 5-, 10-, 15-, or 20-min rest interval did not alter RPP, RMP, or FI compared to a baseline condition without back squats.^17^ However, there were differences between the control condition and the participant's maximal performance, independent of rest interval duration. Thus, the authors suggested that the optimal rest duration between a conditioning activity and the Wingate test is highly individualized, which may have contributed to the null effect.^17^ French et al. reported isometric knee extensions failed to enhance 5-s Wingate test performance.^13^ Doma et al. demonstrated a 2.2 % increase in mean power output during a 30-s Wingate after overloaded cycling and a 10-min rest interval, with no alterations in peak power at the 5- or 10-min rest interval conditions.^9^ Considering the vast mixed results, potential movement specificity disconnect between the stimulated muscle group and the targeted action may have contributed to the lack of significance in these outcomes.

The lack of change in CMJ, as indicated in the present study, has also been observed previously. Five repetitions of tuck jumps did not enhance CMJ height after a 7-, 8-, and 9-min rest interval in male professional academy soccer players.^35^ Three sets of five tuck jumps had no benefit on subsequent CMJ peak power performed immediately after, and 4-, 8-, and 12-min after the tuck jumps.^37^ These findings illustrate that a plyometric conditioning activity, in the form of tuck jumps, may have no positive effects on CMJ performance, which adds to the mixed results of plyometrics as conditioning activities. Additionally, light load jump squats had no effect on CMJ height.^15^ In contrast, plyometrics consisting of ankle hops, hurdle hops, and drop jumps (40 total contacts) enhanced CMJ height after a 1-, 3-, and a 5-min rest interval by ∼4.0 % compared to a CMJ performed prior to the plyometric.^36^ In the present study, the only statistical finding for the CMJ was that males had a greater CMJ height than females collapsed across time and condition by approximately 31.3 %, similar to previous reports.^18^^,^^22^ However, a limitation of this study was the use of only the Vertec without more sensitive methodologies, such as force plates that could detect impulse and other variables. The present participants were not classified as athletes, which may limit the generalizability of the findings to competitive athletics, yet they did indicate resistance exercise experience. Therefore, these findings may not be directly transferrable to athletes. The PAPE response can be highly individualized; therefore, the optimal rest interval duration can differ on an individual basis. To best elucidate the effect of plyometric conditioning activities on CMJ performance, future studies should investigate alterations in rest interval duration, the specific plyometric utilized, the exercise volume of the plyometric performed, and participant characteristics (e.g., athletes vs. resistance-trained individuals).

In conclusion, 10-s Wingate performance (RPP and RMP) can be enhanced by a brief series of tuck jumps and 1–5 min rest. With these outcomes in mind, athletes can easily complete a series of tuck jumps in any setting, keeping in consideration the applicability to the targeted movement. For example, tuck jumps may be valuable to enhance track and field sprints. With inconsistencies in methodologies existing in the literature in relation to PAPE and plyometric conditioning activities, caution needs to be exercised when trying to identify the appropriate pair of conditioning activity and desired outcome measure.

## Funding/support statement

No financial or material support of any kind was received for the work described in this article.

## Declaration of competing interest

The author(s) have no conflicts of interest relevant to this article.
